# *In silico *experimental evolution: a tool to test evolutionary scenarios

**DOI:** 10.1186/1471-2105-14-S15-S11

**Published:** 2013-10-15

**Authors:** Bérénice Batut, David P Parsons, Stephan Fischer, Guillaume Beslon, Carole Knibbe

**Affiliations:** 1 Université de Lyon, CNRS, INRIA, INSA-Lyon, LIRIS, UMR5205, F-69621, France; 2 Université de Lyon, CNRS, LBBE, UMR5558, F-69622, France; 3 Université Lyon 1, INRIA, CNRS, LIRIS, UMR5205, F-69622, France

## Abstract

Comparative genomics has revealed that some species have exceptional genomes, compared to their closest relatives. For instance, some species have undergone a strong reduction of their genome with a drastic reduction of their genic repertoire. Deciphering the causes of these atypical trajectories can be very difficult because of the many phenomena that are intertwined during their evolution (e.g. changes of population size, environment structure and dynamics, selection strength, mutation rates...). Here we propose a methodology based on synthetic experiments to test the individual effect of these phenomena on a population of simulated organisms. We developed an evolutionary model - aevol - in which evolutionary conditions can be changed one at a time to test their effects on genome size and organization (e.g. coding ratio). To illustrate the proposed approach, we used aevol to test the effects of a strong reduction in the selection strength on a population of (simulated) bacteria. Our results show that this reduction of selection strength leads to a genome reduction of ~35% with a slight loss of coding sequences (~15% of the genes are lost - mainly those for which the contribution to fitness is the lowest). More surprisingly, under a low selection strength, genomes undergo a strong reduction of the noncoding compartment (~55% of the noncoding sequences being lost). These results are consistent with what is observed in reduced *Prochlorococcus *strains (marine cyanobacteria) when compared to close relatives.

## Background

Comparative genomics has revealed that some species have exceptional genomes, compared to their closest relatives. Testing hypotheses about the evolutionary causes of these atypical trajectories is a challenge, because models of evolution usually used for phylogenetic reconstruction are not always valid in such lineages. Explaining the reductive evolution observed in some bacterial species is one of these challenges. In this situation, the genomic sequence has been strongly reduced, resulting in the loss of genes, metabolic pathways, regulation capacities, etc. Reductive evolution is one of the characteristics of endosymbiotic life [1-4], but endosymbiotic life is not the only situation where genomes have undergone a reductive evolution. Some oceanic bacteria (e.g. *Pelagibacter ubique, Prochlorococcus marinus*) also possess a highly reduced genome [5-7]. Moreover, when compared to known relatives (e.g. *Synechococcus sp*.) these genomes also show a strong reductive history [6-9]. Yet there is almost nothing in common in the environmental conditions of endosymbionts and marine bacteria. Effective population sizes, modes of transmission, resources, and environments are all different. This raises at least two difficult questions: (1) are the causes of genome streamlining similar in both situations and (2) why do such different ways of living produce similar dynamics on the genome? One way to approach these questions is to perform evolutionary experiments [10]. By cultivating bacteria lineages in the lab, one could theoretically modify one evolutionary parameter at a time to observe which ones lead to genome streamlining. Besides, the mutational events that led to genome reduction can be precisely identified [11] and the structure of the reduced sequence can ultimately be compared with the structure of real reduced bacteria to check whether the mode of reduction is similar. Unfortunately, such a research program is almost impossible to perform. First, the environmental conditions in which streamlining occurs are impossible to reproduce in the lab. Second, numerous pressures act simultaneously on real evolving genomes (e.g. repair mechanisms, recombination, direct and indirect selective pressures, etc.) and it is almost impossible to manipulate them one at a time. Moreover, when submitted to new conditions, an organism may react in various ways. For instance, in the Long-Term Evolutionary Experiment initiated in 1988 by Richard Lenski at Michigan State University, half of the replicates rapidly experienced a strong increase in their mutation rates through mutations affecting their repair pathways [12]. In one word, real organisms are far too complex to perform the "pure", fully controlled experiments that would help to test the various hypothetic mechanisms that may cause an atypical genome evolution. One way to perform such pure experiments and to allow practitioners to strictly change one evolutionary parameter at a time is to use synthetic experiments in which the evolving organisms are not real bacteria but rather models of bacteria. In these synthetic experiments (a.k.a. "in silico experimental evolution" [10] or "digital genetics" [13]), simulated organisms compete, reproduce and mutate inside the computer. It is then possible to test evolutionary scenarios and to observe their consequences on the organisms' structure, at the different levels implemented in the simulation (e.g. genome, regulation network, phenotype, population). Obviously, working with simulated - false - organisms is the major drawback of this approach. However, we argue that, on the other hand, it allows for "perfect experiments" where all the characteristics of the organisms are perfectly mastered, as well as the characteristics of the evolutionary process (mutation rates, mutation bias, selection strength...). Moreover, simulations can be repeated as many times as it is necessary to gain statistical power. They can also last for millions of generations, the only limiting factor being the computational load. Finally, during the experiment, all events can be recorded (including those that did not go to fixation), thus enabling a complete analysis of the evolutionary history. These properties make synthetic experiments a valuable - although not perfect - link between phylogenetic approaches of evolution (that can study long to very long time scales but without direct access to the evolutionary process itself) and experimental evolution (that offers a close view of the evolutionary process but that rarely goes over a few thousand generations).

Here, we present both the general principles of these "synthetic experiments" and a specific platform, called aevol, in which the artificial organisms possess a genetic sequence that can be easily compared to real bacterial genomes at the level of the dynamics of gene repertoire and organization on the chromosome. We then illustrate the insights that can be gained through such an approach on a question like reductive evolution, by testing the effect of a specific evolutionary scenario.

## Methods

*In silico *experimental evolution consists in building artificial organisms and letting them reproduce and mutate inside the computer. An artificial "biochemistry" is designed to decode genomes and compute fitnesses, based on the achievement of a computational task. Evolutionary runs are seeded with random genomes, hand-written genomes or evolved genomes from previous runs. At each time step, a part or the whole population is renewed by letting the fittest organisms reproduce - possibly with mutations - and letting other organisms die. Backups of the whole population can be regularly saved on the disk to constitute a "fossil record". Genealogical trees can also be saved to enable reconstruction of the line of descent of the final best organism at the end of the run.

The size of the population, its spatial arrangement, the mutation rates, the rate of sexual recombination, the fitness measure and the strength of selection are chosen at the beginning of the run and some can also be allowed to change during the run. For example, the population can undergo regular bottlenecks followed by expansion, or the mutation rates can be fixed throughout the run or allowed to evolve. Mutations occur randomly and can have fitness effects ranging from lethal to beneficial and including neutral. For a given parameter set, the experiment is usually repeated several times by performing independent evolutionary runs. Contingency can thus be distinguished from necessity by searching for instances of parallel evolution in the repeated runs.

Several formalisms have been proposed to represent the genome. It can be a collection of alleles like in population genetics, but it can also be a computer program, a graph, a string of functional elements, or a sequence of nucleotides (see [10,14] for recent reviews). The task depends on the formalism. For example, when the genome is a computer program, the task can be the achievement of arithmetical or logical operations on numbers given as inputs. When the genome encodes a gene regulatory network, either through a graph or through a string of functional elements, the task can be to reach to predefined target concentrations for specific proteins. We present below a platform for *in silico *experimental evolution that belongs to the "sequence-of-nucleotides" family, where the genome encodes a variable number of genes separated by a variable amount of non-coding DNA, and where the task is the approximation of a mathematical function with a combination of elementary functions encoded by the genes.

### The aevol platform

The aevol (*a*rtificial *evol*ution) platform was designed to study the evolution of the size and organization of bacterial genomes in various scenarios. It comes as a set of C++ command-line programs, including (i) the main program to perform evolutionary runs and (ii) auxiliary programs to prepare the initial population, visualize the state of an evolved population at a given time, or analyze the mutations on the line of descent of an evolved organism. These programs can be run under Linux or MacOS X, with or without graphical output, depending on whether one wants to get a live impression of the evolution or perform a systematic campaign of experiments on a computer cluster. The source code is available at http://www.aevol.fr.

#### Overview

**Figure 1:**
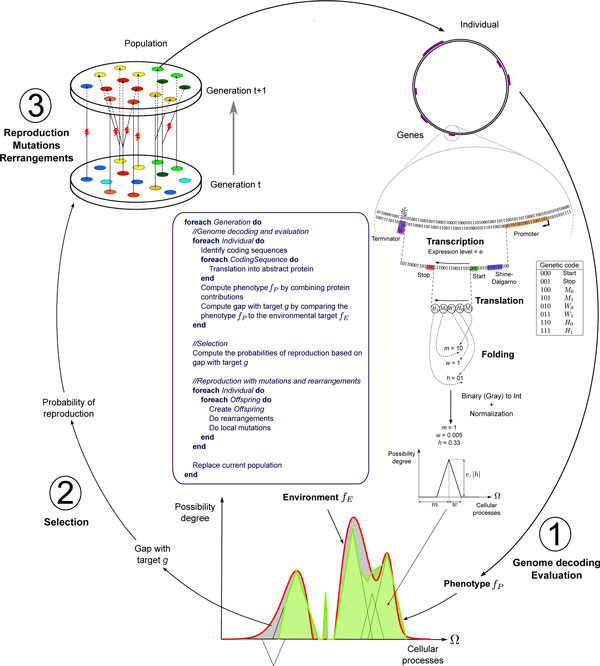
**Graphical representation of the aevol platform**. The underlying algorithm iterates three main steps: (1) genome decoding and evaluation, (2) selection of the best individuals and (3) reproduction with mutations and rearrangements. See the main text for details. The lightning shapes correspond to mutations and rearrangements undergone during reproduction. Cells are colored according to *g*, red cells being those with lowest *g* and blue highest.

The aevol platform simulates the evolution of a population of *N *artificial organisms using a variation-reproduction cycle (Figure 1). In the default setup, the population size *N *is constant over time and is completely renewed at each time step. Each artificial organism owns a circular, double-stranded chromosome, which is actually a string of binary nucleotides, 0 being complementary to 1. This chromosome contains coding sequences (genes) separated by non-coding regions. Each coding sequence is detected by a transcription-translation process (detailed below) and decoded into a "protein" able to either activate or inhibit a range of abstract "cellular processes". The interaction of all proteins yields the set of processes the organism is able to perform. These global functional capabilities constitute here the phenotype. Adaptation is then measured by comparing the phenotypic capabilities to an arbitrary set of cellular processes needed to survive in the environment. At each time step, *N *new individuals are created by reproducing preferentially the best adapted individuals of the parental generation. After that, all individuals from the parental population die. With this "generational" reproduction model, an individual could have several offspring. Thence a generation in aevol corresponds then to many generations in real bacteria. In the experiments presented below, reproduction was strictly asexual. When a chromosome is replicated, it can undergo point mutations, small insertions and small deletions, but also large chromosomal rearrangements: duplications, large deletions, inversions, translocations. Thus mutations can modify existing genes, but also create new genes, delete some existing genes, modify the length of the intergenic regions, modify gene order, etc.

#### Details of the phenotype computation

The phenotype computation starts by searching in both strands for promoter and terminator sequences, delimiting the transcribed regions. Promoters are sequences whose Hamming distance *d *with a pre-defined consensus is less than or equal to *d*_max_. In the experiments presented here, the consensus was 0101011001110010010110 (22 base pairs) and up to *d*_max _= 4 mismatches were allowed. Terminators are sequences that would be able to form a stem-loop structure, as the ρ-independent bacterial terminators do. Here the stem size was set to 4 bases and the loop size to 3 bases. Promoters and terminators delimit the transcribed regions. Note that several promoters can share the same terminator, in which case we obtain overlapping transcribed regions. We assign an expression level $e=1-\frac{d}{1+d_{max}}$ to the transcript, according to the distance *d *between the promoter and the consensus.

Once all the transcribed regions have been localized, we search inside each of them for the initiation and termination signals of the translation process. These signals delimit the coding sequences. The initiation signal is the motif 011011****000 (Shine-Dalgarno-like signal followed by a start codon, 000 here). The termination signal is simply the stop codon, 001 here. Each time an initiation signal is found, the following positions are read three at a time (one codon at a time) until a stop codon is encountered. If no stop codon is found in the transcribed region, no protein is produced. A given transcribed region can contain several coding sequences (overlapping or not), implying that operons are allowed.

To determine the phenotypic contribution of each coding sequence, we use the fuzzy logic framework and the corresponding theory of possibility. We consider an abstract set Ω=[0,1]⊂ℝ of cellular processes that can be performed. A "cellular process" is simply represented by a real number between 0.0 and 1.0. Since  ℝ is an ordered set, some "cellular processes" are closer to each other than to others, just as - in a very informal manner - glucose metabolism can be considered to be closer to lactose metabolism than to DNA repair. Each protein can contribute to or inhibit a subset of Ω, with a variable degree of possibility depending on the cellular process. Formally, the phenotypic contribution of a protein is represented by a mathematical function f:Ω→[0,1], called possibility distribution. For each "cellular process"  x, it defines the degree of possibility f(x) with which the protein can perform  x. We have chosen to use piecewise-linear distributions with a triangular shape (Figure 1). Three parameters are necessary to fully characterize such distributions: the position *m *("mean") of the triangle on the axis, which corresponds to the main cellular process of the protein, the height *H *of the triangle, which determines the degree of possibility for the main process, the half-width *w *of the triangle, which represents the functional scope of the protein and is thus a way to quantify its pleiotropy. Hence the protein can be involved in the "cellular processes" ranging from m-w to m+w, with a maximal degree of possibility for the function at position *m*. The subset of processes the protein affects is thus defined on the interval ]m-w,m+w[⊂Ω. While *m *and *w *are fully specified by the coding sequence, *H *is a composite parameter taking into account both the expression level of the sequence and the intrinsic efficiency of the protein: H=e.|h|, where *e *is the expression level of the transcript and *h *is the efficiency of the protein, coded in the gene sequence as *m *or *w*. Thus, the phenotypic contribution of a given protein is tuned by its primary sequence (*h*), the quality (*e*) of its promoter(s) and possibly variations in gene copy number (concentration effect). As we shall see below, the sign of *h *determines whether the protein contributes to or inhibits the cellular processes ]*m *- *w, m *+ *w*[.

In computational terms, the coding sequence is interpreted as the interlacing of the Gray codes of the three parameters *m, w *and *h *(the Gray code, also known as the reflected binary code, is a variant of the binary encoding where two successive values differ by only one bit, thereby avoiding the so-called Hamming cliffs of the traditional binary code). In more biological terms, the coding sequence is read one code at a time and an artificial genetic code (shown in Figure 1) is used to translate it into the three real numbers *m, w *and *h*. In this genetic code, two codons are assigned to each parameter. For instance, *w *is calculated from the codons W0 = 010 and W1 = 011. All the W codons encountered while reading the coding sequence form the Gray code of *w*. The first bit of the Gray code of w is a 0 (resp. a 1) if the first W codon of the sequence is a W0 (resp. a W1). Hence, if the coding sequence contains $n_{w}$ codons of type W, it encodes an integer comprised between 0 and $2^{n_{W}}-1$. A normalization enables us to bring the value of the parameter in the allowed range specified at the beginning of the simulation. The parameter *w*, which determines the width of the triangle, is normalized between 0 and $w_{max}$, where $w_{max}$ is a parameter defined at the beginning of the simulation. The raw integer value, 1 in our example, is multiplied by $\frac{w_{max}}{2^{n_{W}}-1}$. The values of parameters *m *and *h *are obtained in a similar manner, *m *being normalized between 0 and 1, and *h *between -1 and 1.

The possibility distribution of several proteins - i.e. their triangles - can overlap partially or completely. This means that several proteins can contribute to the same "cellular process". Lukasiewicz's fuzzy operators are used to compute the global functional abilities of the individual. If *f_i _*is the possibility distribution of the *i*-th activator protein (protein with *h *> 0), and *f_j _*the possibility distribution of the j-th inhibitory protein (with *h *< 0), then the phenotype of the individual is represented by the possibility distribution $f_{P}:\text{Ω}\to \left[0,1\right]$ such that $f_{P}\left(x\right)=\text{max}\left(\text{min}\left(\underset{i}{\sum }f_{i}\left(x\right),1\right)-\text{min}\left(\underset{j}{\sum }f_{j}\left(x\right),1\right),0\right)$.

#### Details of the selection step

The environment in which the population evolves is also modeled by a possibility distribution $f_{E}$ on the interval [*0*,*1*]. $f_{E}$ specifies the optimal degree of possibility for each "cellular process" and it can be naught for some processes. This distribution is chosen at the beginning of the simulation and can fluctuate over time if desired (see below). The adaptation of an individual is measured by the gap $g=\int_{0}^{1}\left|f_{E}\left(x\right)-f_{P}\left(x\right)\right|dx$ between its phenotype $f_{P}$ and the target $f_{E}$ ("gap with target"). The probability of reproduction is then $\frac{e^{-kg}}{\sum_{i=1}^{N}e^{-kg_{i}}}$ where *k *is a parameter controlling the strength of selection. The value of *k *determines the steepness of the distribution of the fitness effects of mutations, or, in population genetics terms, the steepness of the distribution of the coefficient of selection *s*. The actual number of offspring of each individual is drawn according to the multinomial law with parameters $\left(N,\left(\frac{e^{-kg_{1}}}{\sum_{i=1}^{N}e^{-kg_{i}}},\frac{e^{-kg_{2}}}{\sum_{i=1}^{N}e^{-kg_{i}}},...,\frac{e^{-kg_{N}}}{\sum_{i=1}^{N}e^{-kg_{i}}}\right)\right)$.

In the experiments reported below, the target distribution $f_{E}$ was built as the sum of three Gaussian functions (see Figure 1). The mean of each of the three bell-shaped functions fluctuated at each time step around their average position, according to an autoregressive process of order 1 with parameters σ and τ: $x_{i}\left(t+1\right)=\bar{x_{i}}+\text{Δ}x_{i}\left(t+1\right)$ with $\text{Δ}x_{i}\left(t+1\right)=\text{Δ}x_{i}\left(t\right)\left(1-\frac{t}{\tau }\right)+\frac{\sigma }{\tau }\sqrt{2\tau -1}\varepsilon \left(t\right)$. In the last equation, the ε(t)~N(0,1) for each Gaussian are independent from one another and normally distributed. σ controls the amplitude of the fluctuations and τ controls the speed at which *x*_i _tends to return to ${\bar{x}}_{i}$.

#### Details of the mutation step

Each time an individual reproduces, its genome is replicated and several types of mutations can occur during this replication. For a point mutation, a random position is changed from 0 to 1 or conversely. For a small insertion (resp. small deletion), a short random sequence (with length uniform between 1 and 6 bp) is inserted (resp. deleted) at a random location. For a large deletion or an inversion, two positions *p*_1 _and *p*_2 _are uniformly drawn on the chromosome and the segment {*p*_1_,..., *p*_2_} is deleted (resp. inverted). For a duplication or a translocation, three positions *p*_1_, *p*_2 _and *p*_3 _are uniformly drawn on the chromosome and a copy of the segment {*p*_1_,..., *p*_2_} is inserted (resp. moved after circularization) at position *p*_3 _in its original orientation.

For each of the seven types of mutation, a per-position rate *u*_type _is chosen at the beginning of the simulation. The mutation algorithm proceeds as follows: when an individual reproduces, we compute the four numbers of rearrangements its genome will undergo. The number of large deletions is drawn from the binomial law *B*(*L, u*_largedel_), the number of duplications from the law *B*(*L, u*_duplic_), and so on. All these rearrangements are then performed in a random order. Once all rearrangements have been performed, we draw the three numbers of local mutations (point mutations, small insertions and small deletions) and we perform all these events in a random order. The genome length can vary throughout this process.

#### Variants

In the default setup presented above and used in this paper, the population is well-mixed, the individuals are asexual, they have a single chromosome, proteins do not regulate gene expression levels of other genes and the breakpoints for rearrangements are drawn uniformly. It is however possible to choose more complicated setups. The breakpoints for the rearrangements can be based on sequence similarity [15]. The individuals can be spatially arranged on a 2D grid and compete locally rather than globally. They can cooperate by producing a public good [16,17]. They can own one or several plasmids and exchange them. Proteins can be allowed to modulate gene expression levels, thereby allowing for the study of gene regulatory network evolution [18].

## Results

In this section, we first present briefly the typical outputs and use of an *in silico *experimental evolution platform like aevol. Then we illustrate more concretely the insights that such simulations can bring by detailing an experiment aimed at testing whether a relaxed selection pressure can lead or not to genome shrinkage.

### Typical use of an in silico experimental evolution platform

The primary outputs of an *in silico *experimental evolution are time series, giving for example the fitness, genome size, gene number at each time step for the current best individual and for the average of the population. Other outputs like genealogical trees can be used to analyze the mutations that occurred in the line of descent of the final best individual (see for example [19]). Additional tests can be performed on this final best individual, like mutagenesis experiments to measure its mutational robustness or the level of epistasis in its genome (see for example [20]).

Distinguishing between fortuitous events and systematic trends can be done by comparing repetitions (runs seeded with different initial sequences), or replay experiments (runs seeded with a backup of a past state of an evolved population but with a different random generator seed). Such backups can also be used to re-run the evolution from a point in the past but with different parameters, to simulate evolutionary scenarios like a sudden decrease in population size or an increase in mutation rates. It is also possible to combine backups of evolved genomes to create mixed populations for competition assays (see for example [21]).

### An example of aevol usage: testing whether relaxed selection alone leads to reductive evolution

Aevol was designed as a tool for testing evolutionary scenarios. Here we show how it can be used for testing different evolutionary conditions that may lead to a reductive genome evolution. Among all possible causes for reductive evolution, we focus here on a reduction in the selective pressures. Note that this test is presented here as a demonstration of the possibilities of aevol. More thorough experiments will have to be performed to test conditions of reductive evolution in the model.

A small effective population size (*N_e_*) is supposed to increase the genetic drift [22,23] and the selective pressures are then reduced on non-essential genes. Because of frequent reproductive bottlenecks, the endosymbionts are supposed to live within populations with small *N_e _*[24] due to host association. Populations of *Prochlorococcus*, on the other hand, are very large [25]. However estimations of *N_e _*are not available and can be considerably lower than the real population size in case *e.g*. of colonization of new niches or of a low recombination rate. No matter why genetic drift may have (hypothetically) increased, such a general relaxation of selection would impact genes differently according to their essentiality.

Using aevol, we tested the effect of a relaxed selection alone, by simulating first a phase of "normal" selection and then a phase of weak selection, without changing the population size. In aevol, the strength of selection is controlled by the parameter *k*, which is used to compute the reproduction probability of each individual given its *g*_i _and the total gap with target of the whole population (see Methods section). In population genetics terms, the parameter *k *influences the coefficient of selection (*s*) of mutations. In aevol, *s *is not explicit as it depends on genes, mutations and phenotype of each individual. For two individuals that differ by only one mutation leading to gaps *g_1 _*and *g_2_, k *determines how the difference between *g_1 _*and *g_2 _*will impact the reproductive success. The higher *k*, the more the difference between *g_1 _*and *g_2 _*impacts the relative reproductive success. Thus, the higher *k *the steeper the distribution of *s *(although the exact distribution remains unknown).

To test the impact of relaxed selection, four simulations with identical parameters (Table 1) were run with populations of 1,000 individuals, starting from a clonal population with a random sequence of 5,000 bases and at least one functional gene. They were run during 300,000 generations with *k* = 750 (control). These four simulations were then replayed between t = 150,000 and t = 300,000 with a lower value for *k *(*k = *250) (scenario).

**Table 1:** Parameter values used for the runs detailed in the Results section

Parameter	Symbol	Value
Population size	*N*	1,000
Size of the initial (random) genome	*L*_init_	5,000 base pairs
Promoter sequence		0101011001110010010110, with up to dmax = 4 mismatches
Terminator sequences		$abcd***\bar{d}\bar{c}\bar{b}\bar{a}$
Initiation signal for the translation		011011****000
Termination signal for the translation		001
Genetic code		See Figure 1
Global set of "cellular processes"	Ω	[0,1]
Maximal pleiotropy of the proteins	*w*_max_	5.10^-3^
Environmental target fluctuates around...	${\bar{f}}_{E}$	See Figure 1
Environmental fluctuations: characteristic time	*τ*	2,500
Environmental fluctuations: standard deviation	*σ*	5.10^-3^
Selection intensity	*k*	750 initially, then 250
Point mutation rate	*u*_point_	5.10^-6 ^per bp
Small insertion rate	*u*_smallins_	5.10^-6 ^per bp
Small deletion rate	*u*_smalldel_	5.10^-6 ^per bp
Large deletion rate	*u*_largedel_	5.10^-5 ^per bp
Duplication rate	*u*_duplic_	5.10^-5 ^per bp
Inversion rate	*u*_inv_	5.10^-5 ^per bp
Translocation rate	*u*_transloc_	5.10^-5 ^per bp
Length of small indels		Uniform law bw. 1 and 6 bp

These parameter values were chosen after preliminary analyses. Some parameters like the structural signals have been shown not to impact the genome structure. The impact of *w_max _*has been studied [30] as well as the impact of mutation rates and particularly rearrangement rates [26]. Here, the mutation rates and *w_max _*values were chosen to obtain a gene density close to bacterial gene density and with enough genes to allow experiments on reductive genome evolution. The intensity and frequency of environmental variations (*σ *and *τ *respectively) are currently under study; *k *is tested here.

**Figure 2:**
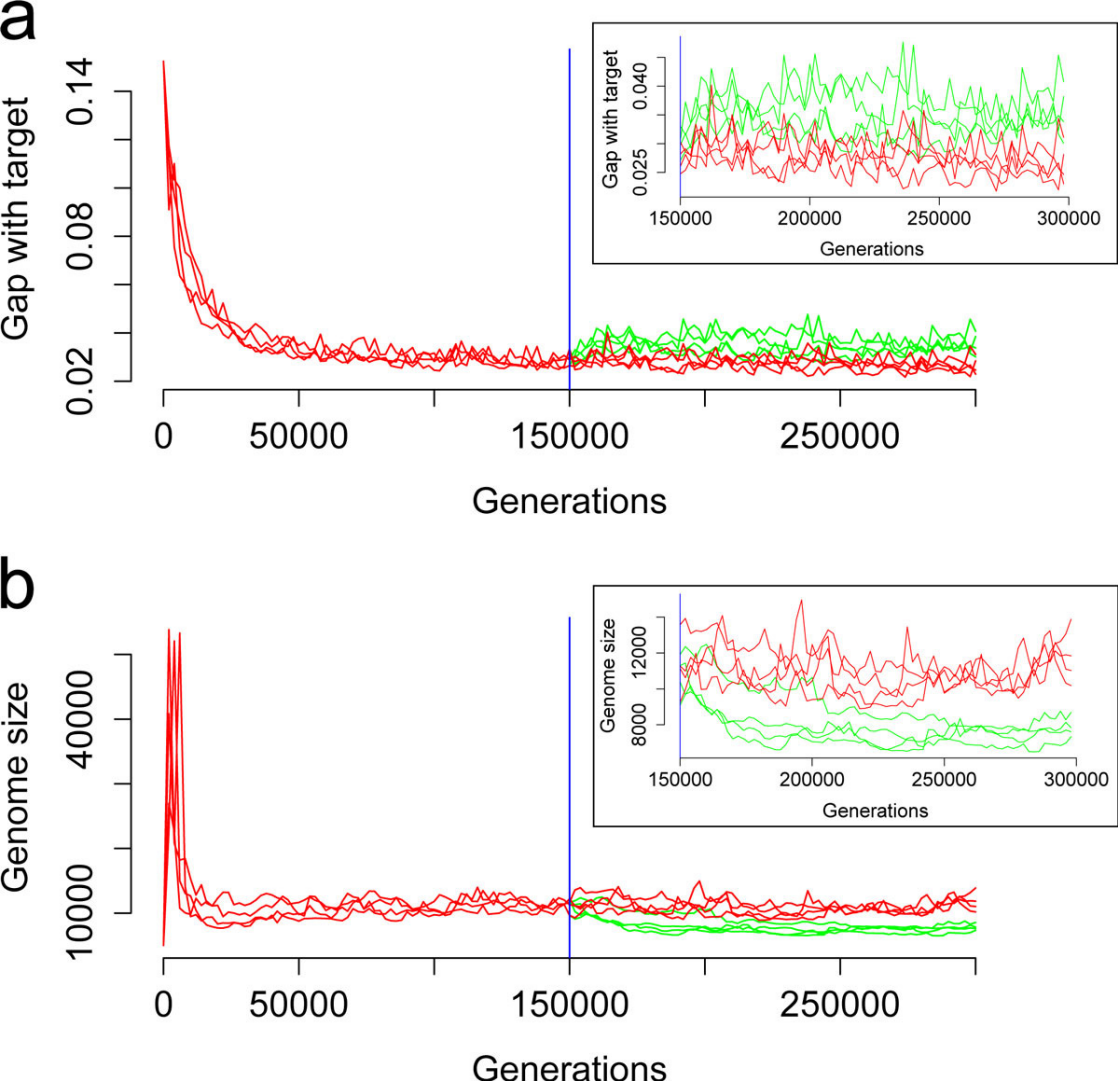
**Gap with target and genome size over time**. The presented data is *g *and genome size for the best individual of the population every 2,000 generations (red: runs with *k *= 750, green: runs with *k *= 250). At t = 150,000, the blue line symbolizes the moment at which *k *is changed in 4 out of 8 simulations. The insets correspond to a zoom from t = 150,000 to t = 300,000.

**Figure 3:**
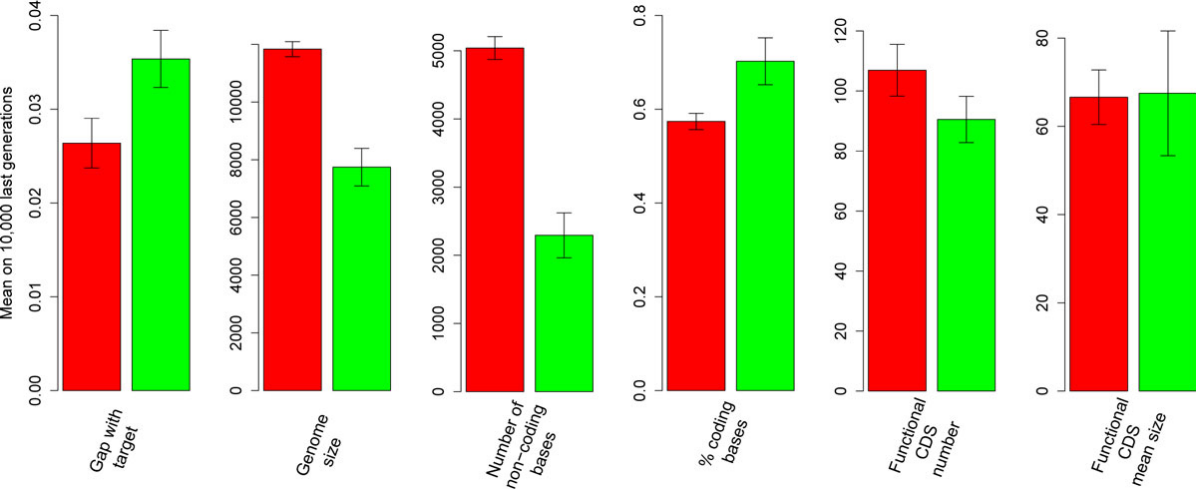
**Several genome architecture characteristics**. For each run, estimates of the genomic characteristics at equilibrium were computed by averaging the values of the best individuals of the last 10,000 generations. Each bar represents the mean of those equilibrium values over the 4 repetitions (red: runs with *k *= 750, green: runs with *k *= 250). The functional CDS are genes involved in cellular processes. The number of noncoding bases corresponds to bases that are not in any RNA with at least one functional gene. The percentage of coding bases is the ratio between the number of bases involved in functional genes and the genome size.

**Figure 4:**
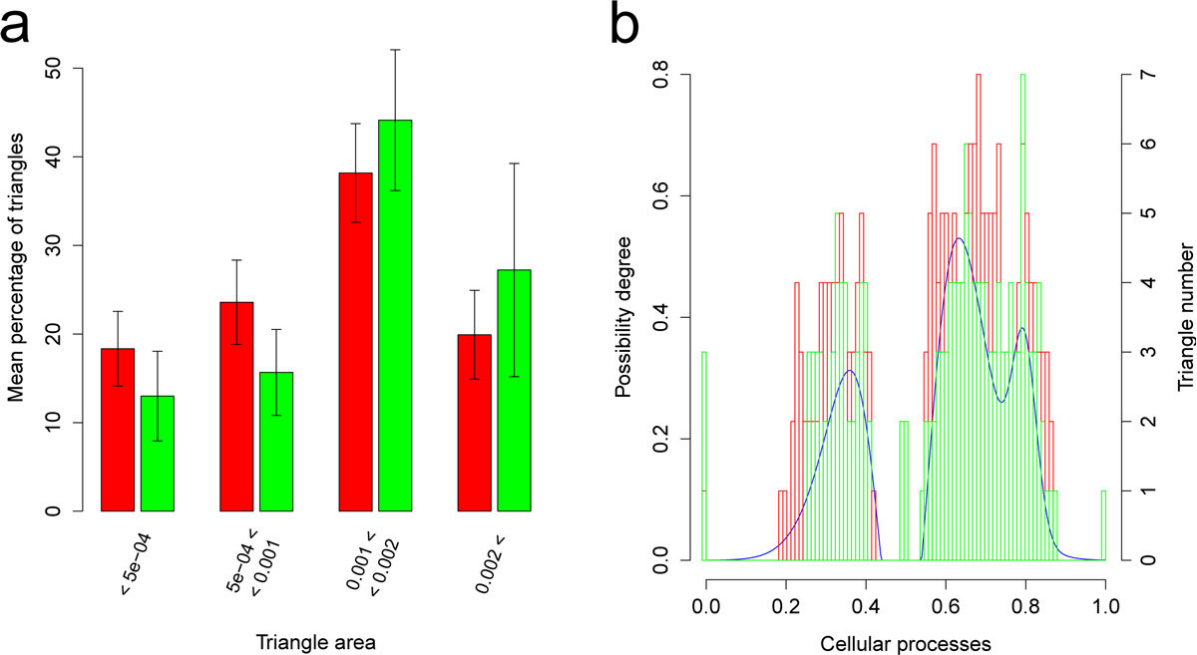
**Distribution of triangle areas and number of triangles per cellular process**. a. The area of a gene's triangle is a proxy for its impact on phenotype and fitness. For each run, the genes of the final best individual were binned into area classes. The red (resp. green) bar plot is the average of the four distributions obtained from the final best individuals of the four runs where *k *= 750 (resp. *k* = 250). b. Distribution of the number of triangles per cellular process for the best individual of one simulation with *k *= 750 in red and one simulation with *k *= 250 in green. Under relaxed selection, the number of triangles per process is reduced.

After 150,000 generations of relaxed selection, the final best individual was less fit than in the control (higher *g*: Figure 2a and Figure 3) because of the loss of functional genes (Figure 3). Genome size had decreased between generations t = 150,000 and t = 200,000 (Figure 2b) leading to a significant difference in genome size at t = 300,000 between the control and the scenario (Figure 2b and Figure 3). As shown on Figure 3, the genomes had lost functional genes but the remaining ones were not shorter. Genomes that had evolved under relaxed selection contained fewer genes with a small contribution to phenotype but more genes with a high contribution (Figure 4a). Actually, under low selection pressure, the "cellular processes" are performed in a simpler way (i.e. fewer triangles but with a higher individual contribution) than under a high selection pressure (Figure 4b). Indeed, reducing *k *from 750 to 250 homogenizes the population in terms of reproduction probabilities. Hence the proportion of quasi-neutral mutations/rearrangements increases: genes having a small impact on the phenotype are more at risk of being lost by genetic drift than others. This leads to a reorganization of the phenotype which is then performed by fewer genes having a higher individual impact. Moreover, analyses of RNA sequences after 300,000 generations show that, under a reduced selection, genomes carry operons with slightly more genes than under a high selection pressure: under high selection (*k *= 750), the mean number of functional CDS per RNA is equal to 1.99 ± 0.41. It increases to 2.50 ± 0.34 under relaxed selection (*k *= 250).

Although the coding sequences have been impacted by the reduction of the selection pressure, the major cause of genome reduction with relaxed selection is the loss of non-coding bases (Figure 3). This is a good example of the surprises that can arise with this experimental approach, which makes no *a priori *assumptions about what selection acts upon. In the model, non-coding DNA has strictly no influence on the phenotype and is thus not expected to be affected by the strength of selection. Yet, it clearly is.

This phenomenon is caused by an indirect selection for an appropriate level of mutational variability. Indeed, we have shown in a previous study [26] that the successful genomes at the evolutionary time scale are those that produce a little more than one "neutral offspring", that is to say, offspring without mutations or with only neutral mutations. In more formal terms, $F_{\upsilon }W\approx 1$, where $W=N\frac{e^{-kg}}{\sum_{i=1}^{N}e^{-kg_{i}}}$ is the expected number of offspring and $F_{\upsilon }$ is the probability for an offspring to have either no mutations or only neutral ones. This reflects the indirect selection of a trade-off between replication accuracy and evolvability, which acts along with the direct selection of adapted individuals. Under relaxed selection (low values of *k*), the reproductions are more equally shared and the values of *W *in the population are more homogenous. Ill-adapted individuals thus "take" reproductions from the fit ones. Since *W *no longer has much of an effect, the successful lineages will be those where $F_{\upsilon }$ is increased. Reducing the amount of non-coding DNA is a way to increase $F_{\upsilon }$, because non-coding DNA is mutagenic for the genes it surrounds [26]. Indeed, large intergenic sequences flanking a gene or a gene cluster enhance its probability of being lost through a large deletion.

According to the hypothesis of Lynch and Conery [22], genetic drift impacts genome size with accumulation of non functional DNA by the spread of selfish DNA elements, or any DNA sequence that may eventually interfere with the organism's fitness. This relation is broadly accepted for eukaryotes but not for prokaryotes [23,27]. Endosymbionts like *Buchnera aphidicola *with a high level of genetic drift kept a low percentage of non-coding DNA despite many gene losses [28,29]. It thus appears that bacteria where the efficacy of selection is low have their genome reduced drastically. In support of this idea, efficacy of selection seems to correlate positively with genome size in bacteria [23]. The genome shrinkage we observed under relaxed selection would generally support this theory. However, the loss of noncoding DNA observed in the simulations is more in agreement with the high coding ratio observed in *Pelagibacter ubique *genome [5] or in the reduced *Prochlorococcus *strains (Table 2). Endosymbionts, on the contrary, exhibit approximately the same proportion of noncoding DNA as other prokaryotes ([29] and Table 2). Thus, while relaxed selection could have played a role in reductive evolution, it alone cannot account for all the patterns of reductive evolution observed in both endosymbionts and marine bacteria.

**Table 2:** Genome characteristics of *Escherichia coli, Buchnera, Prochlorococcus *and simulations.

Genome characteristics	Free-living vs Endosymbionts			*Prochlorococcus*			Experimental evolution		
	***E. coli***	***Buchnera ***		**Non reduced**	**Reduced**		***k *= 750**	***k *= 250**	

**Genome size (bp)**	5.13 × 10**^6 ^**±0.2 × 10**^6^**	0.586 × 10**^6 ^**±0.09 × 10**^6^**	**-89%**	2.545 × 10**^6 ^**±0.19 × 10**^6^**	1.726 × 10**^6 ^**±0.07 × 10**^6^**	**-32%**	11,836 ±261	7,746 ±652	**-35%**
**Coding bases (bp)**	4.54 × 10**^6 ^**±0.14 × 10**^6^**	0.508 × 10**^6 ^**±0.1 × 10**^6^**	**-89%**	2.136 × 10**^6 ^**±0.22 × 10**^6^**	1.54 × 10**^6 ^**±0.05 × 10**^6^**	**-28%**	6,797 ±320	5,453 ±729	**-20%**
**Non coding bases (bp)**	5.882 × 10**^5 ^**±0.74 × 10**^5^**	0.774 × 10**^5 ^**±0.18 × 10**^5^**	**-87%**	4.112 × 10**^5 ^**±0.26 × 10**^5^**	1.849 × 10**^5 ^**±0.27 × 10**^5^**	**-55%**	5,038 ±168	2,293 ±329	**-55%**
**% coding bases**	88.6 ±1	86.5 ±4	**-2%**	83.2 ±2.3	89.3 ±1.2	**+7%**	57.4 ±1.7	70.2 ±5	**+22%**
**Gene number**	5,095 ±166	545 ±98	**-89%**	2,733 ±570	1,987 ±153	**-27%**	107 ±9	91 ±8	**-15%**
**Average gene length (bp)**	896.2 ±4.7	931 ±14.2	**+4%**	797 ±85.6	769.1 ±36.3	**-4%**	66.6 ±6.2	67.5 ±14.1	**+1%**

Genomic data (genome size, genes) was obtained from the NCBI database. Coding and non coding bases, gene length and percentage of coding bases are computed using custom Python scripts for *Escherichia coli *(O157:H7 str. Sakai, 55989, E24377A, O127:H6 str. E2348/69, S88), *Buchnera aphidicola *(str. APS, Cinara tujafilina, Bp, Sg), non reduced *Prochlorococcus *(MIT9303, MIT9313), reduced *Prochlorococcus *(str. AS9601, MIT9211, MIT9215, MIT9301, MIT9312, MIT9515, NATL1A, NATL2A, CCMP1375, CCMP1986) and simulations with *k *= 750 and *k *= 250. For the simulated genomes the real values are irrelevant and cannot be compared with real organisms. To compare with the evolutionary scenario, we show the percentages of reduction (resp. increase) of the different structural parameters. While the reductive evolution of *Buchnera *has equally affected all genomic compartments, in *Prochlorococcus *as well as in aevol the reductive evolution mainly affected the non-coding sequences, resulting in an increase in the coding proportion and a moderate loss of genes.

## Conclusion

In this paper, we have presented a methodology based on synthetic experiments to test hypotheses about atypical evolutionary trajectories like reductive genome evolution. The aevol model, specifically designed to study the evolution of gene repertoire and gene organization in bacterial genomes, was presented. As an illustration of the possibilities of the methodology, we used aevol to let populations of the same artificial "species" evolve under different selective strengths. The simulations show that the genome reduction by loss of some genes and of large segments of noncoding DNA observed e.g. in *Prochlorococcus*, could be explained by a relaxed selection. However, our results are less consistent with patterns of reduced genomes in endosymbionts (more drastic reduction but same proportion of coding bases in endosymbionts than in free-living relatives - Table 2). Alternative explanations are thus required in this case. Indeed, the present methodology could be used to test other hypotheses like a reduced population size, an elevated mutation rate or a stabilization of the environment. Another perspective of the present study is to study the dynamics of genome reduction. Indeed, as shown in Figure 2, the reduction occurs in the first 5,000 generations after the lowering of selection strength. Although a generation in aevol corresponds to multiple bacterial generations, this dynamics of change is rapid. It could be very interesting to study which kind of mutational events have been fixed in this interval and compare them to early events that occurred in endosymbionts and in *Prochlorococcus*. Indeed, although there are no spontaneous mutational biases in the simulation, it is clear that, during these 5,000 generations, a fixed bias should be observed in the winning lineage. Whether this bias will be more visible on rearrangements or on small indels could shed light on the reduction process and on the way genes and noncoding sequences have been lost due to the lowering of selection pressure.

## Competing interests

The authors declare that they have no competing interests.

## Authors' contributions

GB, CK and DPP designed the model. BB, GB and CK designed the experiments. BB, GB, CK and SF analyzed the results. BB, GB, DPP and CK wrote the paper.

## Declarations

This research and publication were funded by the Centre National de la Recherche Scientifique (interdisciplinary programs PEPS and PEPII) and the Institut Rhône-Alpin des Systèmes Complexes (IXXI).

This article has been published as part of BMC Bioinformatics Volume 14 Supplement 15, 2013: Proceedings from the Eleventh Annual Research in Computational Molecular Biology (RECOMB) Satellite Workshop on Comparative Genomics. The full contents of the supplement are available online at http://www.biomedcentral.com/bmcbioinformatics/supplements/14/S15.
